# Genotoxicity Evaluation of Irrigative Wastewater from Shijiazhuang City in China

**DOI:** 10.1371/journal.pone.0144729

**Published:** 2015-12-14

**Authors:** Xuehui Liu, Longmei Tang, Lixue Yang, Xiaolin Zhang, Liqin Wang, Fengxue Yu, Yi Liu, Qing Chen, Dianwu Liu

**Affiliations:** 1 Department of Occupational and Environmental Health, School of Public Health, Hebei Medical University, Zhongshan East Road 361, Shijiazhuang, 050017, Hebei, China; 2 Department of Epidemiology and Hygienic Statistics, School of Public Health, Hebei Medical University, Zhongshan East Road 361, Shijiazhuang, 050017, Hebei, China; 3 Department of Physical and Chemical Inspection, Shijiazhuang Center for Disease Control and Prevention, Likang Street 3, Shijiazhuang, 050011, Hebei, China; 4 Division of gastroenterology, The Second Hospital of Hebei Medical University, Heping West Road, Shijiazhuang, 050000, Hebei, China; 5 Department of Toxicology, School of Public Health, Hebei Medical University, Zhongshan East Road 361, Shijiazhuang, 050017, Hebei, China; 6 Department of Reproductive Medicine, First Affiliated Hospital of the Medical College, Xi'an Jiaotong University, Yanta West Road 76, Xi’an, 710061, Shanxi, China; NIEHS/NIH, UNITED STATES

## Abstract

In the present study, the wastewater sample collected from the Dongming discharging river in Shijiazhuang city was analysed using both chemical analysis and biological assays including the *Salmonella* mutagenicity test, micronucleus test and single-cell gel electrophoresis. Chemical analysis of the sample was performed using gas chromatography mass spectrometry and inductively coupled plasma mass spectrometry. The *Salmonella* mutagenicity test was performed on *Salmonella typhimurium* TA97, TA98, TA100 and TA102 strains with and without S9 mixture. The mice received the wastewater *in natura* through drinking water at concentrations of 25%, 50%, and 100%. One group of mice was exposed for 2 consecutive days, and the other group of mice was exposed for 15 consecutive days. To establish the levels of primary DNA damage, single-cell gel electrophoresis was performed on treated mouse liver cell. The concentrations of chromium and lead in the sample exceeded the national standard (GB20922-2007) by 0.78 and 0.43-fold, respectively. More than 30 organic compounds were detected, and some of the detected compounds were mutagens, carcinogens and environmental endocrine disrupters. A positive response for *Salmonella typhimurium* TA98 strain was observed. Mouse exposure via drinking water containing 50% and 100% of wastewater for 15 consecutive days caused a significant increase of MN frequencies in a dose-response manner. Mouse exposure via drinking water containing 50% and 100% of wastewater for 15 consecutive days caused a significant increase of the Olive tail moments in a dose-response manner. All the results indicated that the sample from the Dongming discharging river in Shijiazhuang city exhibited genotoxicity and might pose harmful effects on the local residents.

## Introduction

To alleviate the shortage of water resources, wastewater irrigation is a widespread practice with a long tradition in the arid and semi-arid regions of the world, especially in developing countries such as China, Mexico, Peru, Egypt, Lebanon, Morocco, India and Vietnam[1]. Globally, approximately 20 million ha of land is reported to be irrigated with wastewater, and at least 10% of the world's population is estimated to consume foods produced by irrigation with wastewater [2]. Farm irrigation with wastewater is reported to exhibit both beneficial and harmful effects [3–4]. Wastewater contains large amounts of nitrogen and phosphorus, which reduces or eliminates the need for supplementary fertilisation for crop growth. However, lots of researches in and abroad have confirmed that a variety of xenobiotics are present in wastewater. These xenobiotics include lead, chromium, cadmium, polychlorinated biphenyls, phthalates, polycyclic aromatic hydrocarbons, organochlorine pesticide and heterocyclic compounds, to name a few[5–8]. The application of wastewater on agricultural land may cause accumulation of metals and persistent organic chemicals in soils and agricultural products, which can potentially harm human and animal health. The contamination of soils and crops due to wastewater irrigation are widely reported in countries such as Germany, France and India [4, 9]. Metals such as cadmium and lead can be sequestered in the soils and absorbed by crops, which serve as the transmission route in the human chain. The persistent organic contaminants accumulated in soil can also be transferred through the food chains and cause adverse effects on human health after long-term exposure[3].

China has a long history of using wastewater for irrigation since the 1940s [10]. The wastewater currently used for farm irrigation in China is mostly untreated and of poor quality. A survey in 1994 found that approximately 85% of the wastewater used for farm irrigation did not meet the nation’s standards for reuse [11]. With urban development, the presence of comprehensive wastewater collection and treatment systems has increased gradually. However, the treatment rate and treatment level of wastewater remain low in China. According to the reports of the Ministry of Environmental Protection of China, the average disposal rate of urban wastewater was 82.3%, and the disposal rate of domestic wastewater was 72.9% in China in 2010. Approximately 45% of the wastewater irrigated areas in China were seriously contaminated with heavy metals [12].

Shijiazhuang City is one of the major wastewater irrigation areas in China, including several areas located in south of the city with a total area covering approximately 438.8 km^2^. Approximately 3.04 x 10^8^ m^3^ of wastewater, which is mainly industrial wastewater and domestic wastewater, is annually discharged through the Dongming discharging channel in Shijiazhuang city. The sources of the wastewater include textile mills, pharmaceutical factories, machinery, electronics, chemical plants, food processing plants, and domestic wastewater from household. The primary pollutants are chlorides, lead, chromium and arsenic [13]. To study the effects of wastewater irrigation on human health, two retrospective studies on the cause of death in wastewater irrigation areas were performed by the Department of Epidemiology and Hygienic Statistics, School of Public Health, Hebei Medical University [14, 15]. Both of the studies found that all-cause standardised mortality and cancer-standardised mortality in a wastewater irrigation area were significantly higher compared with the control area. The results of these studies showed that wastewater irrigation might be an important factor leading to the increased cancer mortality of people living in wastewater irrigation areas. Based on the results of the epidemiological surveys, it is important to evaluate the possible genotoxicity of the irrigative wastewater to effectively manage wastewater irrigation.

Many studies have focused on the genotoxicity of industrial wastewater and domestic wastewater [16–19], while fewer studies have focused on the genotoxicity of irrigative wastewater. The aim of the present research was to investigate the genotoxicity of the irrigative wastewater from Shijiazhuang city in China. More than 200 short-term genotoxicity and mutagenicity assays have been developed for screening potentially carcinogenic chemicals. In summary, most assays were able to detect carcinogens or noncarcinogens with an efficiency of approximately 70% compared with the outcome of 2-year cancer bioassays [20]. Because no single genetic assay can cover all the genetics endpoints, a battery of genetic assays should be used to evaluate the genotoxicity of xenobiotics.

A battery of genotoxicity assays with different genetic endpoints was used in this study: the *Salmonella* mutagenicity test (TA97, TA98, TA100 and TA102; with or without metabolic activation) for base mutation, the micronucleus (MN) test with polychromatic erythrocytes (PCEs) in mice bone marrow for chromosome damage and single-cell gel electrophoresis (SCGE) assays with mice hepatocytes in vivo for primary DNA damage. SCGE assays enable the detection of single-strand and double-strand DNA breaks and apurinic sites [21–24]. The assay is based on the quantification of DNA damage in an electric field and is increasingly used by genetic toxicologists to test individual chemicals and environmental samples.

## Materials and Methods

### 2.1 Reagents and chemicals

Chemicals were obtained from the following sources: glucose-6-phosphate, L-histine, D-botine, nicotinamide adenine dinucleotide phosphate (NADP), dimethyl sulphoxide (DMSO) and XAD-2 resins were obtained from Sigma Chemical Co.; low melting-point agarose was obtained from Biotech; cyclophosphamide (CP) was obtained from Jiangsu Heng Rui Medical Co., Ltd.; phenobarbitol sodium was obtained from Beijing Shuang He Medical Co., Ltd. The chemicals used as positive controls in the *Salmonella* mutagenicity test were supplied by the Center for Disease Prevention and Control of Hebei Province in China. Other solvents and chemicals were of analytical grade.

### 2.2 Sample collection and chemical analysis

Wastewater samples were collected directly upstream (114°31′E, 37°56′N), midstream (114°35′E, 37°51′N) and downstream (114°59′E, 37°41′N) of the Dongming discharging river from Shijiazhuang city during spring (20 April, 2010). No specific permissions were required for these locations. Wastewater samples were collected using an automatic sampling device in a time-proportional manner over a period of 24 h and were stored in precleaned brown glass flasks. The composite sample was filtered with an 11.0 μm pore-size filter and sterilised through a 0.22 μm Millipore filter. The composite sample was stored at -20°C in the dark for chemical analyses and bioassays. Ten liters of the composite sample was extracted using solid-phase extraction with XAD-2 resins according to a previous method [25]. The extracts were redissolved in 1 ml of dimethyl sulfoxide (DMSO). The wastewater samples were collected following the standard method of chemical analysis [26]. Chemical analysis of the extract was performed using gas chromatography mass spectrometry (GC-MS) (Agilent 6890/5973N, USA)). The metal concentrations in the composite sample were determined using inductively coupled plasma mass spectrometry (ICP-MS) (Agilent 7500a, USA).

### 2.3 The Salmonella mutagenicity test

To assess the mutagenicity of the sample, the *Salmonella* mutagenicity test was performed using the standard plate-incorporation method and the preincubation procedure as described [27, 28]. A set of test strains, TA97, TA98, TA100 and TA102, were supplied by the Center for Disease Prevention and Control of Hebei Province in China. A mixture containing 0.1 ml of the sample with different dilution concentrations and 0.1 ml of the test strain was initially incubated at 37°C for 20 min in culture tubes. If metabolic activation was required, 0.5 ml of an S9 mixture was added. The mixture was added to a tube containing 2 ml of top agar with 0.5 mM biotin-histidine. The tube was gently vortexed and poured onto a minimal glucose plate. After incubation at 37°C for 48 h in the dark, the number of revertant colonies was counted and compared to the number of spontaneous revertant colonies on solvent control plates. Four dilution concentrations were examined, including 25 μl, 50 μl, 75 μl, and 100 μl /plate. The samples were tested with or without an S9 mixture. The S9 mixture was prepared from livers of Sprague-Dawley rats pretreated with a polychlorinated biphenyl mixture (Aroclor 1254) and was used in the assay to simulate the metabolic activation occurring in the liver of eukaryotes. Negative and positive controls were also conducted at the same time. Distilled water served as a negative control. Diagnostic mutagens, including 9-fluorenone (0.2 μg/plate), sodium azide (2.5 μg/plate), methylsulfonic methylester (30 μl/plate), 2-aminoflurene (10 μg/plate) and 1,8-dihydroxyanthraquinone (50 μg/plate) were served as positive control chemicals. Triplicate plates were performed for each dose, and the assay was repeated twice.

The sample was considered to be a mutagen when (a) the number of revertant colonies in the assay was at least twice the number of revertant colonies in negative control and (b) a dose-response increase in the number of revertants colonies was observed for one or more strains.

### 2.4 Micronucleus test

Approximately 4-week-old Kunming mice weighing between 20 and 25 g were supplied by the Division of Laboratory Animals of Hebei Medical University. The mice were acclimated for a period of 1 week before the beginning of the experiments. The animals were maintained in a room under controlled conditions of temperature (22±2°C), humidity (50±10%), and a 12 h light/dark cycle. The mice were fed a standard rodent pellet diet (purchased from the Division of Laboratory Animals, Hebei Medical University). During the acclimatisation period, the animals were observed once daily. Mice were accepted for the study upon absence of disease as demonstrated by good physical condition.

Mice were randomly divided into five groups according to weight with 10 animals per group with five males and five females. In a preliminary toxicity assay, neither death nor clinical signs were observed in mice at the maximum recommended volume of 20 ml/kg.bw by gavage. Three dilution concentrations (25%, 50%, and 100%) were performed and administered ad libitum by drinking water. One group of mice was exposed for 2 consecutive days, and the other group of mice was exposed for 15 consecutive days. Distilled water administered to the negative group ad libitum by drinking water for the same period. The positive control group received an intraperitoneal injection of cyclophosphamide (40 mg/kg). At the end of the experimental period, mice were deprived of food for 24 h and prepared for the experimental procedure. The animals were sacrificed by an overdose of CO_2_. At the time of necropsy, the breastbone of each mouse was collected and cleaned of the surrounding muscle tissue. The bone marrow fluids were squeezed out with a hemostat, dropped in the fetal calf serum on one end of the clean slide and mixed carefully. At least two thin smears were prepared by pulling bone-marrow fluids behind a cover glass held at a 45° angle. All the slides were air-dried, fixed in methanol for 10 min, and stained according to the method developed by Schmid et al. [29, 30]. These slides were coded for blind analysis. At least 1000 polychromatic erythrocytes per animal were scored for the presence of micronuclei under immersion objective (1000×) using an Olympus BH-2 microscope. The following criteria were applied for the identification of micronuclei: no connection with the main nucleus, same color and intensity as the main nucleus and an area smaller than one-third of the main nucleus. The ratio of PCE/NCE was also determined by counting a total of 1000 erythrocytes.

A compound can be considered as a mutagen if the induced MN frequencies were statistically significant compared with those induced by the negative control, and a dose-response increase in the number of MN frequencies was observed. This study was carried out in accordance with the Guidelines of Animal Experiments from the Committee of Medical Ethics, Ministry of Health of China that seeks to minimize both the number of animals used and any suffering that they might experience. The protocol was approved by the Ethics Committee for Animal Experiments of Hebei Medical University (approval number: HEBMU -2010-03; approval date: March 25, 2010). Studies comply with Animal Research: Reporting In Vivo Experiment guidelines (S1 Checklist).

### 2.5 SCGE

Kunming mice were randomly divided into five groups according to weight with 8 animals per group, with four males and four females. Three groups received the samples with three dilution concentrations (25%, 50%, and 100%) ad libitum by drinking water for 15 consecutive days. The negative group received distilled water for the same period of time, and the positive group controls received an intraperitoneal injection of phenobarbital sodium (140 mg/kg), which can induce primary DNA lesions [31]. The water intake was recorded every day, and the water intake of each mouse was calculated. At the time of necropsy, a liver-cell suspension with a cell density of 10^4^ ~ 10^5^/ml was prepared. Cell viability was determined using trypan blue dye exclusion [32]. The number of trypan blue negative cells was considered to be the number of viable cells and was superior to 95%. The slides were prepared using the conventional comet assay method. The slides were immersed in a cold, freshly prepared lysing solution. The slides were protected from light and maintained at 4°C for 1 h and placed in electrophoresis buffer at 4°C for 30 min to allow the DNA to unwind before electrophoresis. Electrophoresis was performed at 25 V and 300 mA for 20 min in the dark at room temperature. After electrophoresis, the slides were neutralised in Tris 400 mM (pH 7.5), rinsed three times in distilled water, and left to dry overnight at room temperature. The dry slides were stained according to the method described by Santos et al. [33]. For each animal, 50 cells were evaluated. The Olive tail moments (OTMs, Olive tail moment = percent of DNA in the tail × the distance between the center of gravity of DNA in the tail and the center of gravity of DNA in the head) were observed using the CASP comet analysis software [34].

### 2.6 Statistical analysis

Values were expressed as the mean ± SD. The dose-response relationships were analysed by Spearman correlation using SPSS 18.0. The statistical analysis was performed by one-way ANOVA for the analysis of MN frequencies; the OTMs data of the SCGE were analysed using the nonparametric Kruskal–Wallis test followed by a post-hoc multiple-comparison test. A medullar toxicity analysis involving the PCE/NCE ratio was statistically analysed by Student’s t-test, and *P*< 0.05 was considered statistically significant for the assays.

## Results

### 3.1 Chemical analysis

The following concentrations of heavy metals were measured in the composite sample: Pb, 0.285 mg/l; Cr, 0.178 mg/l; Mn, 0.128 mg/l; Ni, 0.047 mg/l; Be, 0.00044 mg/l; As, 0.028 mg/l; and Cd, 0.002 mg/l. More than 30 organic compounds were detected by GC-MS, including phthalates, heterocyclic compounds, polycyclic aromatic hydrocarbon compounds, aniline compounds, etc. The main organic pollutants are shown in Table 1.

**Table 1:** Main organic pollutions in the irrigative wastewater taken from the Dongming discharging river in Shijiazhuang city determinated by GC-MS.

Name	Molecular formula	Molecular weight
Mono(2-ethylhexyl) phthalate	C_16_H_22_O_4_	278.34
Dibutyl phthalate	C_16_H_22_O_4_	278.34
Dihexyl phthalate	C_20_H_30_O_4_	334.21
Diisobutyl phthalate	C_16_H_22_O_4_	278.15
Phenylacetic acid	C_8_H_8_O_2_	136.05
Dipropyl phthalate	C_14_H_18_O_4_	250.19
Di(2-ethylhexyl)phthalate	C_24_H_38_O_4_	390.56
2-(Methylthio)- benzothiazole	C_8_H_7_NS_2_	181.00
1-Methylfluorene	C_14_H_12_	180.09
Benzothiazole	C_7_H_5_NS	135.01
N-methyl-N-phenyl- Formamide	C_8_H_9_NO	135.07
Indole	C_8_H_7_N	117.06
N-(2-Naphthyl)aniline	C_16_H_13_N	219.28
Benzylidene malonaldehyde	C_10_H_8_O_2_	160.05

### 3.2 The Salmonella mutagenicity test

The results from the *Salmonella* mutagenicity test are shown in Table 2. With and without an S9 mixture, the number of revertant colonies of the TA98 strain induced by the samples of different dilution concentrations was more than twice the negative control. With and without the S9 mixture, the number of revertant colonies of TA97, TA100, and TA102 strains induced by the samples with different dilution concentrations was less than twice the negative control. With and without the S9 mixture, there was no significant dose-response relationship with the number of revertant colonies of TA97, TA100, and TA102 strains induced by the samples with different dilution concentrations. Significant dose-response relationships were observed with the number of revertant colonies of the TA98 strain (*r*_s_ = 0.976, *P* = 0.000; *r*_s_ = 0.954, *P* = 0.000), and the dose-response relationship curves could be fitted well with the equations: ŷ = 34.833+113.133x and ŷ = 42.833+124.133x.

**Table 2:** The Salmonella bioassay of the irrigative wastewater taken from the Dongming discharging river in Shijiazhuang city (n = 6).

	TA97 (-S9)		TA97 (+S9)		TA98 (-S9)		TA98 (+S9)	
Dose (μl/plate)	MI^a^	Revertant colonies	MI^a^	Revertant colonies	MI^a^	Revertant colonies	MI^a^	Revertant colonies
NC^b^	1	97.2±10.2	1	100.7±14.7	1	30.2±2.3	1	30.5±1.4
25	1.2	116.0±15.7	1.1	111.2±13.2	2.3^d^	67.5±7.3*	3.0 ^d^	90.0±14.4*
50	1.2	119.7±15.6	1.2	121.8±7.0	3.2^d^	97.2±4.1*	3.4 ^d^	102.3±12.4*
75	1.2	118.0±10.3	1.3	126.8±5.3	3.7^d^	111.7±7.6*	4.5 ^d^	137.3±4.7*
100	1.3	129.0±6.4	1.3	130.0±7.2	5.0^d^	150.0±7.2*	5.3 ^d^	163.2±9.3*
PC	21.5^d^	2078.5±118.0	12.9^d^	1274.5±108.8	98.2^d^	2950.7±83.3	85.9^d^	2618.5±268.1
	TA100 (-S9)		TA100 (+S9)		TA102 (-S9)		TA102 (+S9)	
Dose (μl/plate)	MI^a^	Revertant colonies	MI^a^	Revertant colonies	MI^a^	Revertant colonies	MI^a^	Revertant colonies
NC^b^	1	116.3±6.6	1	120.0±7.3	1	221.3±11.9	1	220.0±8.8
25	1.1	132.3±10.3	1.1	134.8±8.0	1.1	238.3±11.6	1.1	242.3±12.7
50	1.1	130.0±4.7	1.1	133.5±11.0	1.1	250.8±7.8	1.2	263.8±10.0
75	1.1	130.0±7.1	1.2	144.8±13.7	1.2	258.3±12.0	1.2	263.5±11.0
100	1.2	139.3±5.9	1.1	131.3±8.8	1.1	242.7±7.4	1.1	242.7±7.4
PC^c^	20.3^d^	2368.3±206.9	12.4^d^	1475.5±41.5	9.1^d^	2005.3±120.7	5.3 ^d^	1158.3±163.0

^a^: Mutagenic index (MI): number of revertant colonies induced in the sample/number of spontaneous revertant colonies in the negative control.

^b^: Negative control (NC) for all strains: sterile distilled water

^c^: Positive controls (PC) in experiments: Without S9: 0.2 μg/plate 9-fluorenone was used for strain TA97 and TA98, 2.5 μg/plate sodium azide for strain TA100, 30 μl/plate methylsulfonic methylester was used for strain TA102; With S9: 10 μg/plate 2-aminoflurene was used for strain TA97, TA98, and TA100; 50 μg/plate 1,8-dihydroxyanthraquinone was used for strain TA102

^d^: MI was more than twice that of the negative control.

*: Significant dose-response relationships were observed

### 3.3 Micronucleus test

**Fig 1:**
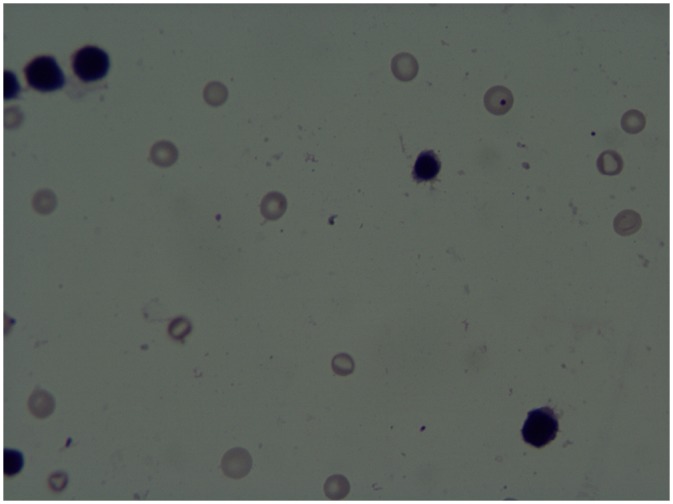
Micronucleus of mice bone polychromatic erythrocytes induced by the irrigative wastewater in Shijiazhuang city.

The micronucleus are shown in Fig 1. The results from the MN test are presented in Table 3. The MN frequencies of the PCEs in mouse bone marrow induced by the samples with different dilution concentrations for two consecutive days were not significantly different from the MN frequencis induced by the negative control. After the mice were exposed to the samples for 15 consecutive days, the MN frequencies of the PCEs in mouse bone marrow induced with the concentrations of 50% and 100% were significantly increased, and a dose-response relationship was observed (ŷ = 0.003+0.007x, *r*_s_ = 0.814, *P* = 0.000). The sample did not affect the proliferation of normal bone marrow erythrocytes, and medullar toxicity was not observed.

**Table 3:** Micronucleus frequencies of PCEs in mouse bone marrow induced by the irrigative wastewater taken from the Dongming discharging river in Shijiazhuang city (n = 10).

	2 consecutive days exposure		15 consecutive days exposure	
Dose	MN (‰±SD)	PCE/NCE ($\bar{\text{x}}$ ±SD)	MN (‰±SD)	PCE/NCE ($\bar{\text{x}}$ ±SD)
NC ^a^	3.40±1.35	1.03±0.07	3.30±1.16	1.02±0.06
25%	3.80±1.48	1.03±0.11	4.00±1.41	1.01±0.07
50%	4.10±1.37	1.00±0.10	7.60±2.17**	1.03±0.07
100%	4.50±1.08	1.00±0.12	9.40±2.27**	0.96±0.06
PC ^b^	17.70±2.00**	0.91±0.08**	16.20±2.35**	0.89±0.06**

^a^: Negative control, Sterile distilled water

^b^: Positive control, cyclophosphamide (40 mg/kg)

** *P*<0.01 versus negative control

### 3.4 SCGE

**Fig 2:**
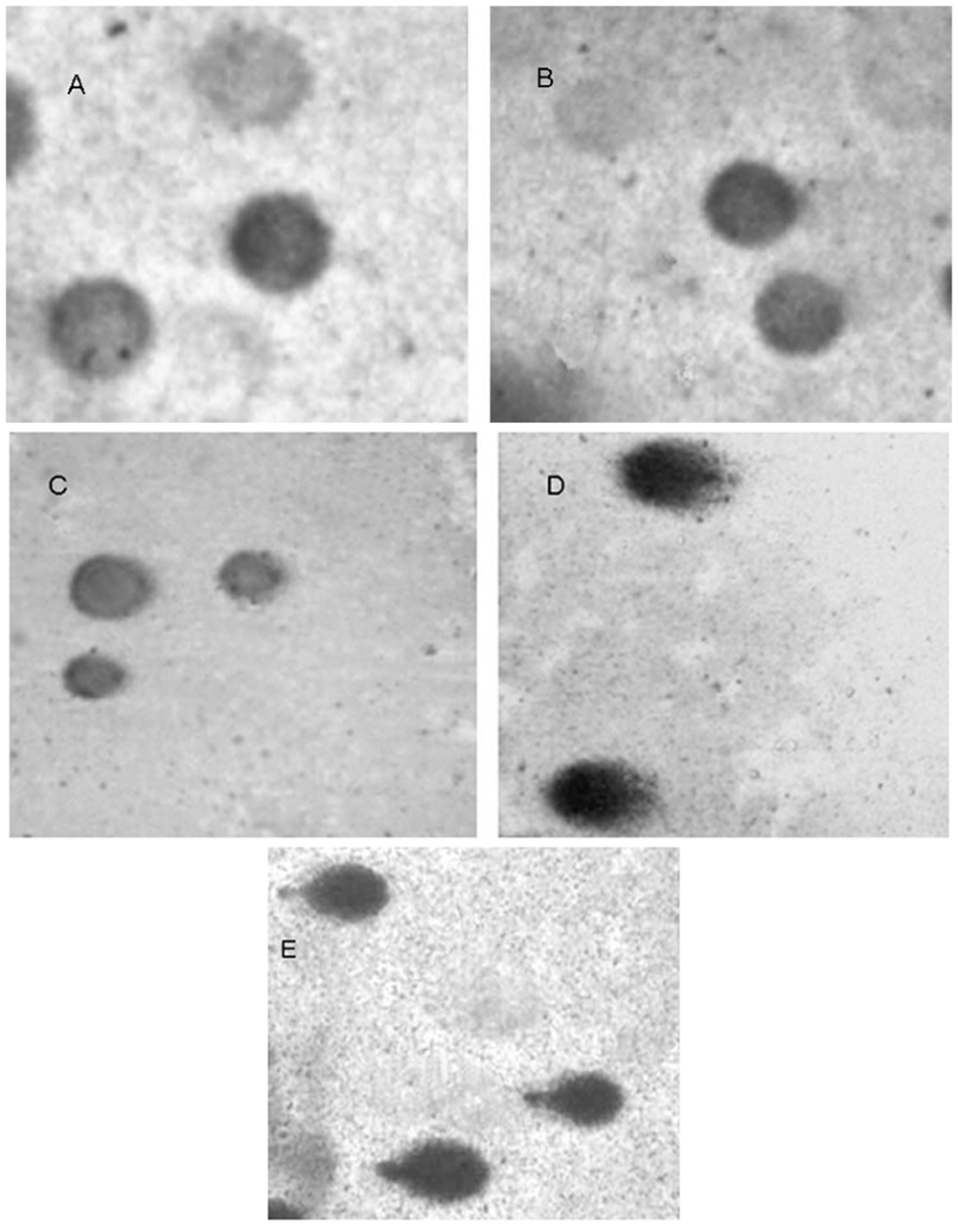
The result of single-cell gel electrophoresis. (A): negative control; (B)25% wastewater; (C) 50% wastewater; (D) 100% wastewater; (E) positive control.

The figures from SCGE are shown in Fig 2 (A for negative control group; B for 25% wastewater group; C for 50% wastewater group; D for 100% wastewater group; E for positive control group). The results from SCGE are presented in Table 4. After the mice were exposed to the sample for 15 consecutive days, the OTMs of the mouse hepatocytes induced with the concentrations of 50% and 100% were significantly increased compared with the negative control, and a dose-response relationship was observed (ŷ = 0.298+4.055x, *r*_s_ = 0.905, *P* = 0.000). No significant difference was observed concerning the body weight and water intake.

**Table 4:** Results from SCGE in mouse hepatocytes induced by the irrigative wastewater taken from the Dongming discharging river in Shijiazhuang city (n = 8, $\bar{\text{x}}$±SD).

Dose	Body weight (g)	Water intake^c^ (ml/day)	Olive tail moment
NC ^a^	34.41±3.00	5.68±0.66	0.65±0.09
25%	32.30±3.57	6.30±0.51	0.68±0.10
50%	33.45±3.61	6.44±0.49	2.58±0.18**
100%	33.01±3.65	5.96±0.70	4.38±0.58**
PC ^b^	34.37±2.92	6.18±0.47	20.18±1.30**

^a^: Negative control, Sterile distilled water

^b^: Positive control, phenobarbital sodium (140 mg/kg)

^c^ Water intake (ml/day) for each mouse

** *P*<0.01 versus negative control

## Discussion

Many national and international studies have focused on the genotoxicity of urban wastewater, industrial wastewater, river and drinking water [16–18, 35, 36]. In these studies, two main methods of sample pretreatment were commonly adopted: organic pollutants may be enriched by solid-phase extraction or liquid-liquid extraction methods to improve the sensitivity of the test substance, or the water sample may be sterilised through a Millipore filter (0.22 μM) and directly used for biological detection. The organic extracts obtained by solid-phase extraction methods commonly induced genetic damage in vivo and in vitro [36–38]. The water samples were concentrated before biological detection for the first method, which may reduce the environmental relevance of the biological exposure concentrations. Each enrichment method can concentrate parts of the composition in the water sample, and some pollutants might be lost during the enrichment process. Other contaminants such as heavy metals were present in wastewater in addition to organic pollutants. Therefore, the organic extracts, which were obtained in the enrichment process and served as the test substance, were not suitable. Therefore, the second sample pretreatment method was recommended because the results from the genotoxicity test using this method were more realistic and reliable. In the study by Durgo et al. [39], the wastewater sample was sterilised through a Millipore filter (0.22 μM), and the results of SCGE indicated significant DNA damaging potential for human leukocytes. Wastewater samples from an oncological ward of the general hospital of Vienna in Austria filtered with an 11.0 μm filter and a 0.22 μm filter were tested using the SCGE assay with primary rat hepatocytes. A significant and dose-dependent induction of DNA damage (up to two-fold over the background) was observed [40]. In the present study, the irrigative wastewater sample from Shijiazhuang city was filtered with an 11.0 μm filter and sterilised with a 0.22 μm Millipore filter.

Two main methods have been used for studies on wastewater. One method uses chemical analysis of the main composition, and the other method uses biological methods (such as SCGE) to detect the total genotoxicity. For research on environmental samples, chemical analysis and biological methods are equally important. The chemical analysis shows the composition of the environmental samples (qualitative analysis) and the concentration of each compound (quantitative analysis), and the biological assays shows the total toxicity on the organisms. Combining biological assays with chemical analyses is a good method for research on environmental samples and contributes to the prevention and control of environmental pollution.

The major metals in the wastewater sample were analysed by ICP-MS. The concentrations of chromium and lead in the sample exceeded the national standards (GB20922-2007) by 0.78 and 0.43-fold, respectively. Positive results for chromium were observed in nine mutation experiments including the *Salmonella* mutagenicity test, SCGE, MN assay, sister chromatid exchange assay, etc. [41, 42]. American researchers found that a cumulative hexavalent chromium exposure showed a strong dose-dependent relationship for lung cancer in a cohort study of 2,357 workers in chromate production facilities [43]. Several studies on the genotoxicity of lead acetate were conducted in rodents using the MN test, and the results showed an increase in MN frequencies [44–47].

The GC-MS analysis of the organic extracts in the composite sample identified more than 30 organic compounds, and some of the compounds were mutagens, carcinogens and environmental endocrine disrupters. Dibutyl phthalate, diisobutyl phthalate, and mono (2-ethylhexyl) phthalate, which are mutagens, were identified in the samples [48, 49]. Some organic pollutants that are not considered to be mutagens may generate mutagenic effects after metabolic activation in the body, such as di(2-ethylhexyl)-phthalate, benzothiazole, and N-(2-Naphthyl)aniline [50–52].

Chemical analysis of complex mixtures offers limited information concerning biological toxicity. Based on the chemical analysis, a battery of genotoxicity assays with different genetic endpoints were used in the research to evaluate the biological toxicity. The combined use of these three bioassays with different genetic endpoints increases the confidence level of genotoxicity estimation. The results of the *Salmonella* mutagenicity test showed that direct and indirect frameshift-type mutagens were present in the sample. Furthermore, the sample could induce DNA damage in a dose-dependent manner in mice hepatocytes in vivo. With increasing exposure duration, the results of the MN test in PCEs of mouse bone marrow changed from negative to positive. Our results were consistent with the findings of earlier investigations. The MN test collaborative research group (CSGMT) reported MN test results of 11 test substances and found that the MN frequencies of multiple exposures were higher compared with a single exposure in most cases, and individual results changed from negative to positive [53]. Genotoxicity can be a consequence of long-term exposure to low levels of chemicals and can exhibit a hereditary and delayed-onset nature that may lead to major consequences at the population level [54]. None of the chemicals detected in the chemical analysis were present at concentrations individually cause genotoxicity; therefore, the potential for component interactions (additive or synergistic) was likely the cause of the genotoxicity of the total sample.

In summary, all the results indicated that the samples from the Dongming discharging river in Shijiazhuang city exhibited genotoxicity and might pose potential harmful effects on the local residents. To standardise the wastewater irrigation management and to maximise the protection of the local residents’ health, the wastewater emissions of lead, chromium and organic pollutants from the upstream factories should be strictly controlled. Wastewater treatment plants should improve the wastewater treatment rate and enhance the removal efficiency of heavy metals and organic pollutants.

## Conclusions

In the present study, the wastewater collected from the discharging river in Shijiazhuang city was analysed using both chemical analysis and biological assays. The concentrations of chromium and lead in the sample exceeded the national standards (GB20922-2007) by 0.78 and 0.43-fold, respectively. More than 30 organic compounds were detected, and some of the compounds were mutagens, carcinogens and environmental endocrine disrupters. The present results from the biological assays conformed that the irrigative wastewater sample exhibited genotoxicity by causing base mutation, chromosomal damage and DNA damage. In summary, the results from both the chemical analysis and biological assays imply that the genotoxic chemicals contained in the irrigative wastewater may harm organisms in the ecosystem and humans as a result of accumulation in the food chain. However, the present work is a preliminary report. Further studies are needed to confirm the carcinogenetic risk of irrigative wastewater on humans.

## Supporting Information

S1 Checklist The ARRIVE guidelines checklist. (PDF)
